# Supplementary figures and images for: The mature N-termini of Plasmodium effector proteins confer specificity of export
Source: mBio. 2023 Aug 30;14(5):e01215-23. doi: 10.1128/mbio.01215-23 (PMC10653839; doi:10.1128/mbio.01215-23)

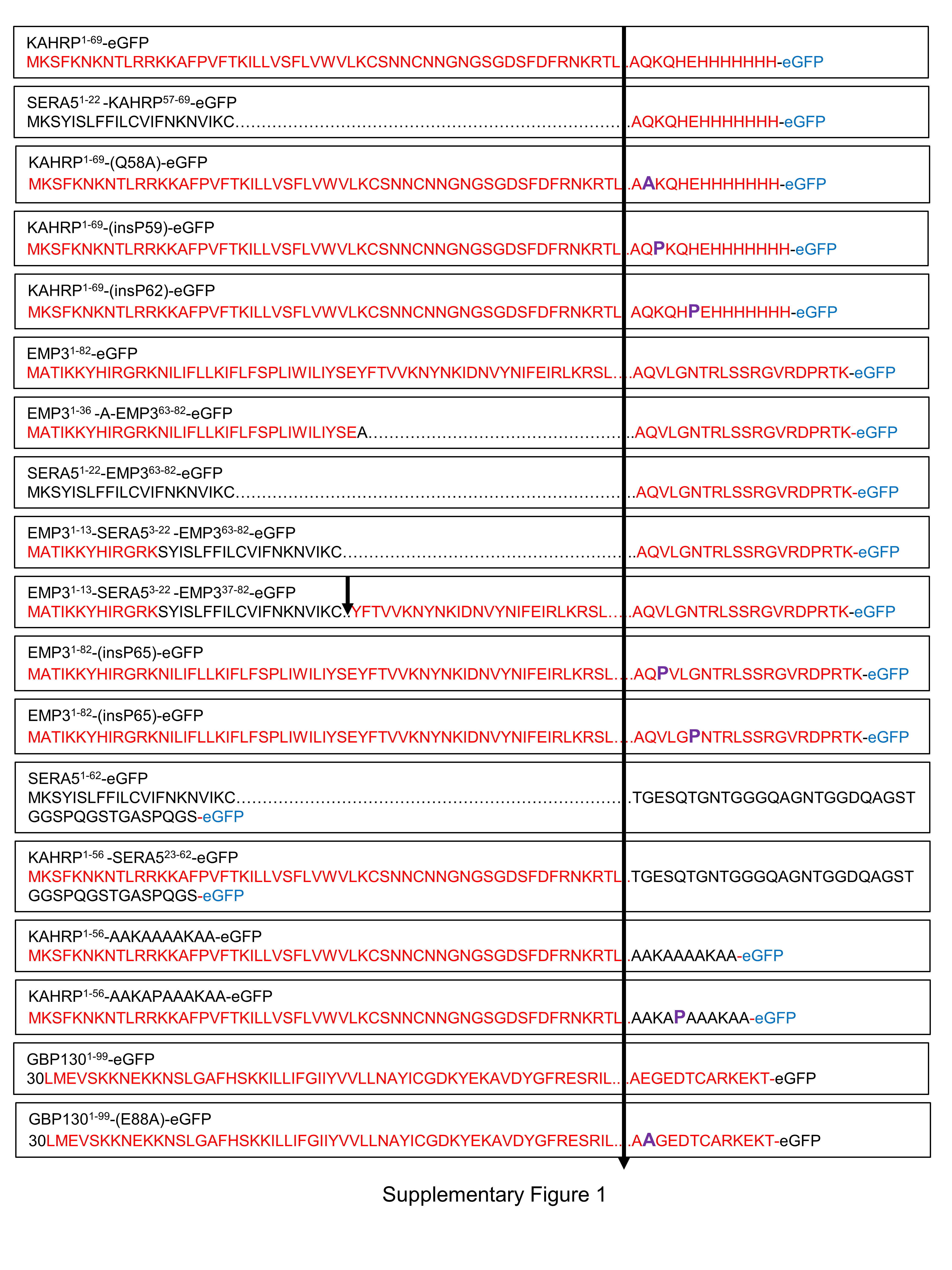

Supplement: Fig. S1 — Full sequence of all the reporters in this study. eGFP sequence is not shown as well as the first 29 residues of GBP130. Bold arrows denote cleavage sites. Sequences from PEXEL proteins are printed in red. Substitutions and insertions are highlighted in bold purple. [file mbio.01215-23-s0001.tif]

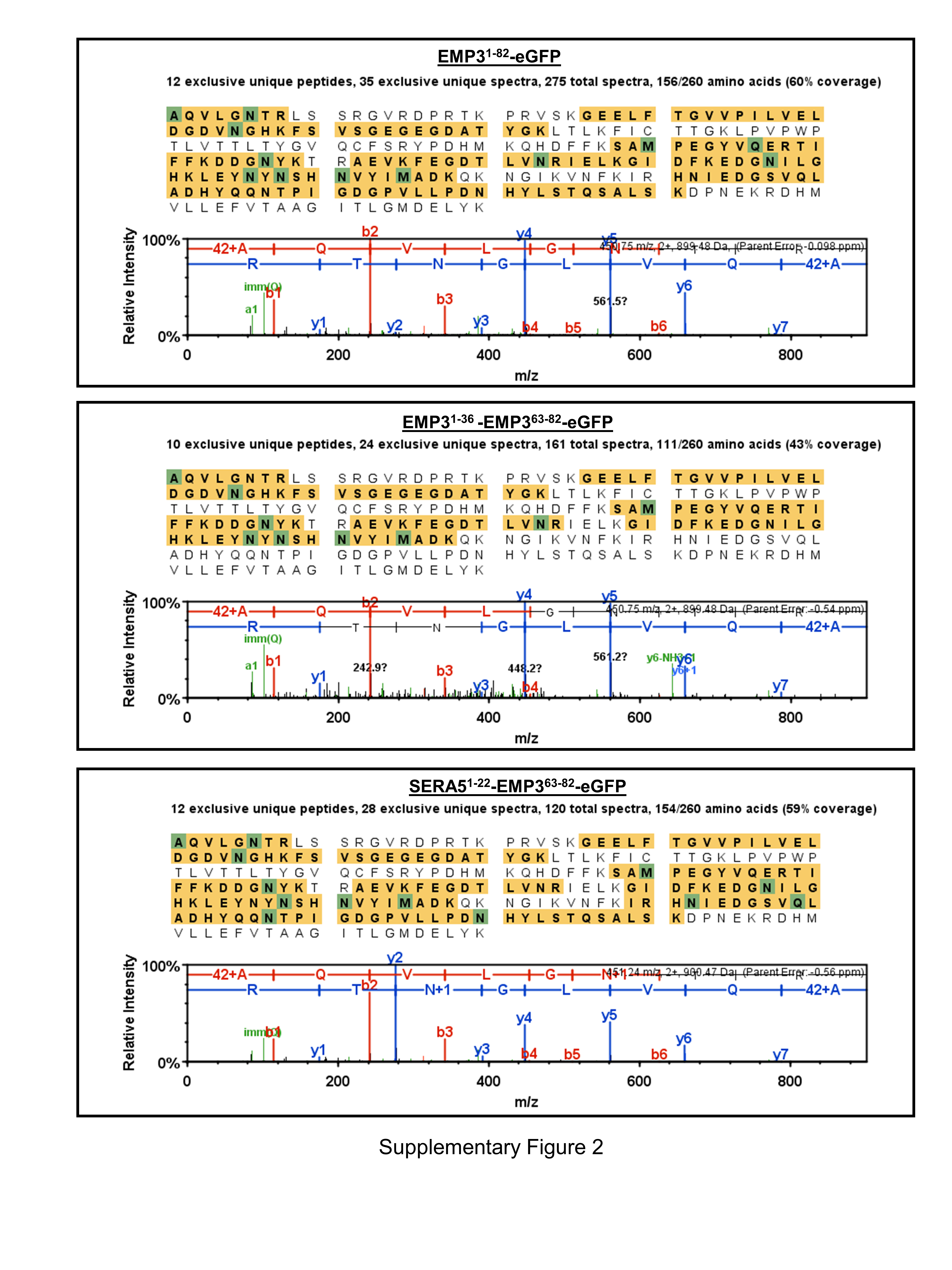

Supplement: Fig. S2 — Coverage map and most N-terminal peptide spectrum of EMP3 reporters. Construct names are on top. The coverage map highlights detected peptides at the 95% threshold. Green shades in the coverage map denote post-translational modification. [file mbio.01215-23-s0002.tif]
