# Supplementary material for: The mature N-termini of Plasmodium effector proteins confer specificity of export
Source: mBio. 2023 Aug 30;14(5):e01215-23. doi: 10.1128/mbio.01215-23 (PMC10653839; doi:10.1128/mbio.01215-23)
Supplement: Tables S1 and S2 — Table S1: list of PEXEL proteins and PV-resident proteins along with their mature N-terminal sequences and AlphaFold structural predictions. These sequences were used to generate the frequency plots shown in Figure 4. For the AlphaFold structural predictions, "a" denotes alpha-helical, "b" denotes beta-sheet, and "c" denotes random coil. Table 2: list of primers used in this study. [file mbio.01215-23-s0003.docx]

Table S1

| PlasmoDB ID | Description | Amino acid sequence (-3 to +10 from the cleavage site) | AlphaFold structure prediction (-3 to +10 from the cleavage site) |
| --- | --- | --- | --- |
| Exported PEXEL proteins | | | |
| PF3D7_1016900 | early transcribed membrane protein 10.3 | RALKDIDRTLEKL | aaaaaaaaaaaaa |
| PF3D7_0831800 | histidine-rich protein II | RLLHETQAHVDDA | aaaaaaaaaaaaa |
| PF3D7_1401300 | proline aminopeptidase | RILCDVIKDGIGA | aaaaaaaaaaaaa |
| PF3D7_0601100 | exported protein family 3 | RILTENNKKWYKK | aaaaaaaaaaaaa |
| PF3D7_0902500 | serine/threonine protein kinase, FIKK family (FIKK9.6) | RYLSEIIIENVTS | aaaaaaaaaaaaa |
| PF3D7_0310400 | parasite-infected erythrocyte surface protein | RLLVDTYKNIKEN | aaaaaaaaaaaaa |
| PF3D7_1001300 | Plasmodium exported protein (PHISTa), unknown function | RTLSEIEKENYTS | aaaaaaaaaaaaa |
| PF3D7_0202200 | EMP1-trafficking protein | RSLAELKKRIGAI | aaaaaaaaaaaaa |
| PF3D7_1476200 | Plasmodium exported protein (PHISTb), unknown function | RCLSEGYLNLYSP | aaaaaaaaaaaaa |
| PF3D7_0301600 | Plasmodium exported protein (hyp1), unknown function | RLLAQSYVFNSRL | aaaaaaaaaaaaa |
| PF3D7_1401200 | Plasmodium exported protein, unknown function | RSLAESYKQYKIN | aaaaaaaaaaacc |
| PF3D7_0902300 | serine/threonine protein kinase, FIKK family (FIKK9.4) | RKLAESNIANERS | aaaaaaaaaaacc |
| PF3D7_0201800 | knob associated heat shock protein 40 | RNLAQTQRNFKSK | aaaaaaaaaaacc |
| PF3D7_1102500 | Plasmodium exported protein (PHISTb), unknown function | RNLYEDELIRKDS | aaaaaaaaaaacc |
| PF3D7_1016600 | Plasmodium exported protein (PHISTc), unknown function | RILSEYQCIENYN | aaaaaaaaaaccc |
| PF3D7_1253000 | gametocyte erythrocyte cytosolic protein | RILSDLEERESES | aaaaaaaaaaccc |
| PF3D7_1016300 | glycophorin binding protein | RILAEGEDTCARK | aaaaaaaaaaccc |
| PF3D7_0113300 | Plasmodium exported protein (hyp1), unknown function | RLLTEYKDTLQIK | aaaaaaaaaaccc |
| PF3D7_0832200.1 | Plasmodium exported protein (PHISTa-like), unknown function | RNLSETESVEHSG | aaaaaaaaaaccc |
| PF3D7_0402000 | Plasmodium exported protein (PHISTa), unknown function | RNLSELQKGNQPC | aaaaaaaaacccc |
| PF3D7_0424500 | serine/threonine protein kinase, FIKK family (FIKK4.1) | RHLTEVWDNNNNN | aaaaaaaaacccc |
| PF3D7_0532600 | Plasmodium exported protein, unknown function | RILKQLEFITLEE | aaaaaaaacccca |
| PF3D7_0731100 | EMP1-trafficking protein | RNLGEKSEEGGIN | aaaaaaaaccccc |
| PF3D7_1001400 | alpha/beta hydrolase, putative | RSLGELLKNPEKR | aaaaaaaaccccc |
| PF3D7_0501000 | Plasmodium exported protein, unknown function | RVLAEQEDQYIRN | aaaaaaaaccccc |
| PF3D7_1001600 | alpha/beta hydrolase, putative | RKLAEALKDDERF | aaaaaaacccaaa |
| PF3D7_0301300 | alpha/beta hydrolase, putative | RYLSETELNKRSD | aaaaaaacccccc |
| PF3D7_0201900 | erythrocyte membrane protein 3 (EMP3) | RSLAQVLGNTRLS | aaaaaaacccccc |
| PF3D7_1101500 | exported protein family 4 | RCLLQHMVEEPFE | aaaaaaacccccc |
| PF3D7_0424700 | serine/threonine protein kinase, FIKK family (FIKK4.2) | RNLSECFRGKSAL | aaaaaaccccccc |
| PF3D7_1301700 | Plasmodium exported protein (hyp8), unknown function | RSLAENQKVETEQ | aaaaaaccccccc |
| PF3D7_0807700 | serine protease DegP | RILNDERNIKITD | aaaaacccccaaa |
| PF3D7_0113700 | heat shock protein 40, type II | RCLAEGNKNFFFN | aaaaacccccccc |
| PF3D7_0500800 | mature parasite-infected erythrocyte surface antigen | RILSETEPPMSLE | aaaaccccccaaa |
| PF3D7_1039000 | serine/threonine protein kinase, FIKK family (FIKK10.2) | RHLSDYSSSNDNL | aaaaccccccccc |
| PF3D7_0936400 | ring-exported protein 4 | RNLSELNLDLRTD | aaacccccccccc |
| PF3D7_1301400 | Plasmodium exported protein (hyp12), unknown function | RLLAEPSSHGSSK | aaacccccccccc |
| PF3D7_0936500 | virulence-associated protein 1 | RFLTQGKVEKDFN | aaacccccccccc |
| PF3D7_0402400 | Plasmodium exported protein, unknown function | RILVEFSNSYYYD | bbbbbcccccccb |
| PF3D7_0202000 | knob-associated histidine-rich protein (KAHRP) | RTLAQKQHEHHHH | ccaaaaaaacccc |
| PF3D7_0936600 | gametocyte exported protein 5 (PHISTc) | RKLSELVDSKSGC | ccaaacccccccc |
| PF3D7_0501200 | parasite-infected erythrocyte surface protein | RTLADFNDMFANQ | cccaaaccccccc |
| PF3D7_1477500 | Plasmodium exported protein (PHISTb), unknown function | RNLSDNEKEKEKE | ccccaaaaaaaaa |
| PF3D7_1001900 | Plasmodium exported protein (hyp16), unknown function | RFLSEPLLEFDTV | cccccccccaaaa |
| PF3D7_0936300 | ring-exported protein 3 | RQLSEPVVEEQDL | cccccccccaaaa |
| PF3D7_0532300 | Plasmodium exported protein (PHISTb), unknown function | RNLCEKKPLNVDM | ccccccccccaaa |
| PF3D7_0401800 | Plasmodium exported protein (PHISTb), unknown function | RNLSEEKAEDDYK | ccccccccccaaa |
| PF3D7_1252700 | Plasmodium exported protein (PHISTb), unknown function | RTLFETNNGNNGY | cccccccccccaa |
| PF3D7_1100800 | Pfmc-2TM Maurer's cleft two transmembrane protein | RMLAQNNTNKKSN | ccccccccccccc |
| PF3D7_1016400 | serine/threonine protein kinase, FIKK family (FIKK10.1) | RCLAEFGSVRNIF | ccccccccccccc |
| PF3D7_0501100.1 | heat shock protein 40, type II | RSLAEFNSGSSRE | ccccccccccccc |
| PF3D7_0730900 | EMP1-trafficking protein | RSLTERKQRNNGK | ccccccccccccc |
| PF3D7_0532400 | lysine-rich membrane-associated PHISTb protein | RKLCERFIYYNPN | ccccccccccccc |
| PF3D7_0113900 | Plasmodium exported protein (hyp8), unknown function | RWLSETSVSYSDE | ccccccccccccc |
| PF3D7_0424600 | Plasmodium exported protein (PHISTb), unknown function | RILSEGQSTDEYS | ccccccccccccc |
| PF3D7_0113200 | Plasmodium exported protein, unknown function | RILADYHNISDTS | ccccccccccccc |
| PF3D7_0102200 | ring-infected erythrocyte surface antigen | RNLYGETLPVNPY | ccccccccccccc |
| PF3D7_0104200 | StAR-related lipid transfer protein | RILKENKEESLET | ccccccccccccc |
| PF3D7_0101800 | Stevor | RLLAQTKNHNPHY | aaaaaacccccca |
| PF3D7_0300800 | RIFIN | RSLCECKLYAQSN | ccccccccccccc |
| PV-resident Proteins | | | |
| PF3D7_1129100 | PV1 | IYGNVVAPKSAEA | ccccccccaaacc |
| PF3D7_1226900 | PV2 | VLGKEEKTMIRTD | acccccccccccc |
| PF3D7_1336700 | PV3 | LKNENKCFCQFTD | aaaaccccccccc |
| PF3D7_0207700 | SERA4 | TTASTTQGGDTDT | ccccccccccccc |
| PF3D7_0207600 | SERA5 | IKCTGESQTGNTG | ccccccccccccc |
| PF3D7_0207500 | SERA6 | LFTQYFIKCEGNK | ccccccccccccc |
| PF3D7_0925900 | Lipocalin | IRASPGNDNNNVN | ccccccccccccc |
| PF3D7_1121600 | EXP1 | SLAEKTNKGTGSG | aaaaacccccccc |
| PF3D7_1024800 | EXP3 | VELKLNNIIYDKN | aaaaaaaaccccc |
| PF3D7_1116800 | HSP101 | VLCAPDNKQEQGK | aaccccaaaaaaa |
| PF3D7_1345100 | Trx2 | VTCTKEVTSTNDD | ccccccccccccc |
| PF3D7_1105600 | PTEX88 | GQCMYKIKQSGDI | ccccccbbbbbbb |
| PF3D7_1436300 | PTEX150 | YCAVQNNGNKSLN | aaaaaaacccccc |

Table S2

| Primer name | Sequence |
| --- | --- |
| KAHRP_F | CGAATAAACACGATTTTTTCTCGAGATGAAAAGTTTTAAGAACAAAAATACTTTGAGGAGAAAG |
| KAHRP_R | cctcgcccttgctgaccctaggATGGTGATGGTGGTGATGGTGTTCATGTTGCTTTTGTGC |
| GBP130_F | CGAATAAACACGATTTTTTCTCGAGATGCGACTTTCTAAAGTATCTGATATTAAATCTACAG |
| GBP130_R | cctcgcccttgctgaccctaggAGTCTTTTCTTTTCGTGCACACG |
| EMP3_F | AAACACGATTTTTTCTCGAGATGGCAACAATAAAAAAATACCATATAAGAGGAAG |
| EMP3_R | GTCGTGGAGTAAGGGATCCTAGAACTAAGcctagggtcagcaagggcga |
| Sera5_F | ACATTTCGAATAAACACGATTTTTTCTCGAGATGAAGTCATATATTTCCTTGTTTTTCATATTG |
| Sera5_R | GAGCAAGTCCACAAGGTAGTcctagggtcagcaagggcga |
| KAHRP_Sera5_mut | CAGAAATAAGAGAACTTTAGCACAAGAAAGTCAAACAGGTAATACAGGAGGAGGTCAAGCAGGTAATACAGGAGGAGATCAAGCAGGTAGTACAGGAGGAAGTCCACAAGGTAGTACGGGAGCAAGTCCACAAGGTAGTcctagggtcagcaag |
| Sera5_KAHRP_mut | AAAAATGTTATAAAATGTACAGGAAAGCAACATGAACACCATCACCACCATCACCATcctagggtcagcaagggc |
| KAHRP Q58A | CCTTCGATTTCAGAAATAAGAGAACTTTAGCAGCAAAGCAACATGAACACCATCACCACCATCAC |
| GBP130_E88A | GATTATGGTTTTAGAGAAAGCAGAATTTTAGCTGCAGGAGAAGATACGTGTGCACGAAAAGAAAAG |
| E2_mut | CCTTTAATATGGATACTAATTTATTCTGAAgccGCCCAGGTTTTGGGGAATAC |
| E3_mut | cgcccttgctgaccctaggATCCCTTACTCCACGACTGCTTAACCTTGTATTCCCCAAAACCTGGGCACATTTTATAACATTTTTGTTAAATATAACACAC |
| E4_mut | GTTATATTTAACAAAAATGTTATAAAATGTGCCCAGGTTTTGGGGAATACAAGGTTAAGC |
| E5_mut | GTTATATTTAACAAAAATGTTATAAAATGTTATTTTACGGTTGTTAAGAATTATAATAAA |
| KAHRP_P59 | TGACTCCTTCGATTTCAGAAATAAGAGAACTTTAGCACAAcctAAGCAAcatGAACACCATCACCACCATCACCATcctagggtcag |
| KAHRP_P62 | CGATTTCAGAAATAAGAGAACTTTAGCACAAAAGCAAcaccctGAACACCATCACCACCATCACCATcctagggtcagcaagg |
| EMP3_P68 | GAAATAAGACTTAAAAGATCATTAGCCCAGGTTTTGGGGccgAATACAAGGTTAAGCAGTCGTGGAGTAAGGGATCCTAGAAC |
| EMP3_P65 | TAATATATTTGAAATAAGACTTAAAAGATCATTAGCCCAGccgGTTTTGGGGAATACAAGGTTAAGCAGTCGTGGAGTAAGGGATC |
| KAHRP  _model_alpha | TCCTTCGATTTCAGAAATAAGAGAACTTTAGCAgctaaggcagctgcagctaaggcagctcctagggtcagcaagggcgaggagctgttc |
| KAHRP_  model_non_alpha | TCCTTCGATTTCAGAAATAAGAGAACTTTAGCAgctaaggcacctgcagctaaggcagctcctagggtcagcaagggcgaggagctgttc |
